# A comprehensive review on phytochemistry, pharmacology, and flavonoid biosynthesis of *Scutellaria baicalensis*

**DOI:** 10.1080/13880209.2018.1492620

**Published:** 2018-12-05

**Authors:** Zi-Long Wang, Shuang Wang, Yi Kuang, Zhi-Min Hu, Xue Qiao, Min Ye

**Affiliations:** State Key Laboratory of Natural and Biomimetic Drugs, School of Pharmaceutical Sciences, Peking University, Beijing, China

**Keywords:** Anti-tumor, anti-viral, Huang-Qin, neuroprotective, traditional Chinese medicine

## Abstract

**Context:***Scutellaria baicalensis* Georgi (Lamiaceae) is a popular medicinal plant. Its roots are used as the famous traditional Chinese medicine Huang-Qin, which is recorded in Chinese Pharmacopoeia, European Pharmacopoeia, and British Pharmacopoeia.

**Objective:** This review comprehensively summarizes research progress in phytochemistry, pharmacology, and flavonoid biosynthesis of *S. baicalensis.*

**Methods:** English and Chinese literature from 1973 to March 2018 was collected from databases including Web of Science, SciFinder, PubMed, Elsevier, Baidu Scholar (Chinese), and CNKI (Chinese). *Scutellaria baicalensis*, chemical constituents, phytochemistry, biological activities, and biosynthesis were used as the key words.

**Results:** A total of 126 small molecules (**1–126**) and 6 polysaccharides have been isolated from *S. baicalensis*. The small molecules can be classified into four structural types, namely, free flavonoids, flavonoid glycosides, phenylethanoid glycosides, and other small molecules. Extracts of *S. baicalensis* and its major chemical constituents have been reported to possess anti-viral, anti-tumor, anti-bacterial, antioxidant, anti-inflammatory, hepatoprotective, and neuroprotective activities. Key steps in the biosynthetic pathways of *Scutellaria* flavonoids have also been summarized.

**Conclusions:** This article could be helpful for researchers who are interested in the chemical constituents, bioactivities, biosynthesis, and clinical applications of *S. baicalensis*.

## Introduction

**Figure 1.**
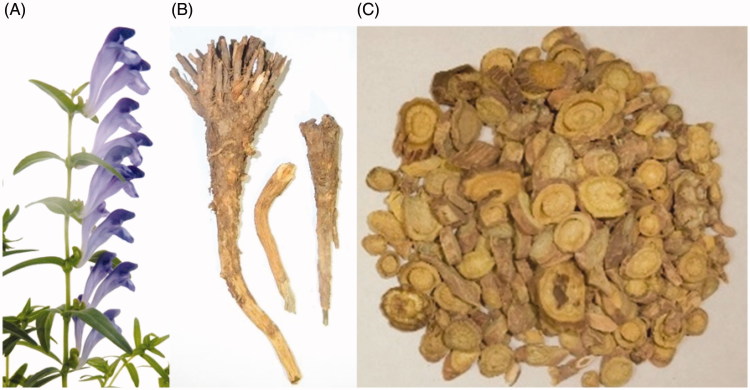
Pictures of the plant (A), TCM crude drugs (B), and TCM prepared slices (C) of *Scutellaria baicalensis*.

The plants of genus *Scutellaria* L. (Lamiaceae) are perennial herbs with around 360 species in the world. Many of these species have medicinal uses (Cantor et al. 2009; Shang et al. 2010; Paton et al. 2016). Among them, the roots of *Scutellaria baicalensis* Georgi are used in China as Huang-Qin (Scutellariae Radix), one of the most popular traditional Chinese medicines (Figure 1). *Scutellaria baicalensis* is widely distributed in North China, Japan, Korea, Mongolia, and Russia (Zhao et al. 2016a; Jiang et al. 2017). Due to its increasing demands in recent years, it is now cultivated on a large scale in Shandong, Hebei, Inner Mongonia, Shanxi, and Gansu provinces of China (Gu et al. 2013). It should be noted that the herb of an allied species, *Scutellaria barbata* D. Don, is used as the Chinese medicine Ban-Zhi-Lian.

In China, *S. baicalensis* has a medicinal history of at least 2000 years. Huang-Qin was first recorded in *Shennong’s Classic of Materia Medica* (Shennong Bencao Jing in Chinese) in around 200 AD. In ancient Chinese language, the character ‘Qin’ means ‘herb for hemostasis’, and ‘Huang’ means yellow color (Li and Li 2017). The Traditional Chinese Medicine (TCM) theory considers Huang-Qin has the functions of clearing heat, eliminating dampness, purging fire, detoxification, hemostasis, and preventing miscarriage. Huang-Qin is now listed officially in Chinese Pharmacopoeia (2015), European Pharmacopoeia (EP 9.0), and British Pharmacopoeia (BP 2018). It is the key component herb for many famous Chinese medicine patent drugs, such as Gegen Qinlian Pills (to treat diarrhea, dysentery, fever, and influenza), Lanqin Oral Liquid (to treat sore throat), Yinzhihuang Granules (to treat jaundice and hepatitis), and Xiongdan Huangqin Eye Drops (to treat conjunctivitis). Flavonoids are the major bioactive chemical constituents of Huang-Qin. Among them, baicalin has been developed into a new drug (Huangqingan Tablets, manufactured by a number of companies including Shanghai Hutchison Pharmaceuticals and Jingfukang Pharmaceutical Group Co. Ltd), and is used to treat acute and chronic hepatitis. The total flavonoids extract of the stems and leaves of *S. baicalensis* has also been developed into a new drug (Huangqin Jingye Jiedu Capsules), and is mainly used to treat sore throat.

Despite the popular clinical use of Huang-Qin, scientific evidences are not adequate to identify the effective chemical components responsible for the versatile biological activities. The quality control of Huang-Qin crude drugs and related patent drugs still needs to be improved, and the medicinal potential of many bioactive compounds of this plant has yet to be explored. A comprehensive review of *S. baicalensis* could be helpful for researchers, manufacturers, and policymakers to obtain a holistic view of this important herbal medicine.

Several review articles are available on the *Scutellaria* genus or *S. baicalensis* (Shang et al. 2010; Zhang et al. 2014; Zhao et al. 2016a; Karimov and Botirov 2017; Cheng et al. 2018). As an increasingly popular herbal medicine, important research progress has been made in recent years. Herein, we comprehensively summarized research literature on phytochemistry, pharmacology, and flavonoid biosynthesis of *S. baicalensis*. English and Chinese literature published during 1973 to March 2018 was collected from databases including Web of Science, PubMed, Elsevier, SciFinder, Baidu Scholar (Chinese), and CNKI (Chinese). *Scutellaria baicalensis*, chemical constituents, phytochemistry, biological activities, and biosynthesis were used as the key words.

## Phytochemistry

To date, a total of 126 small molecule compounds (**1–126**) and 6 polysaccharides have been isolated from *S. baicalensis* Georgi (Figure 2; Table 1). Most of these compounds were obtained from the roots (the Chinese medicine Huang-Qin). A few research groups studied chemical constituents of the aerial part (Ma 2013; Wang HW et al. 2016) and the hairy root cultures (Zhou et al. 1997). The small molecules can be classified into four structure types, i.e., free flavonoids, flavonoid glycosides, phenylethanoid glycosides, and other small molecules. Among them, flavonoids and their glycosides are the major compounds.

Figure 2.Chemical structures of compounds **1–126**.

**Figure:**
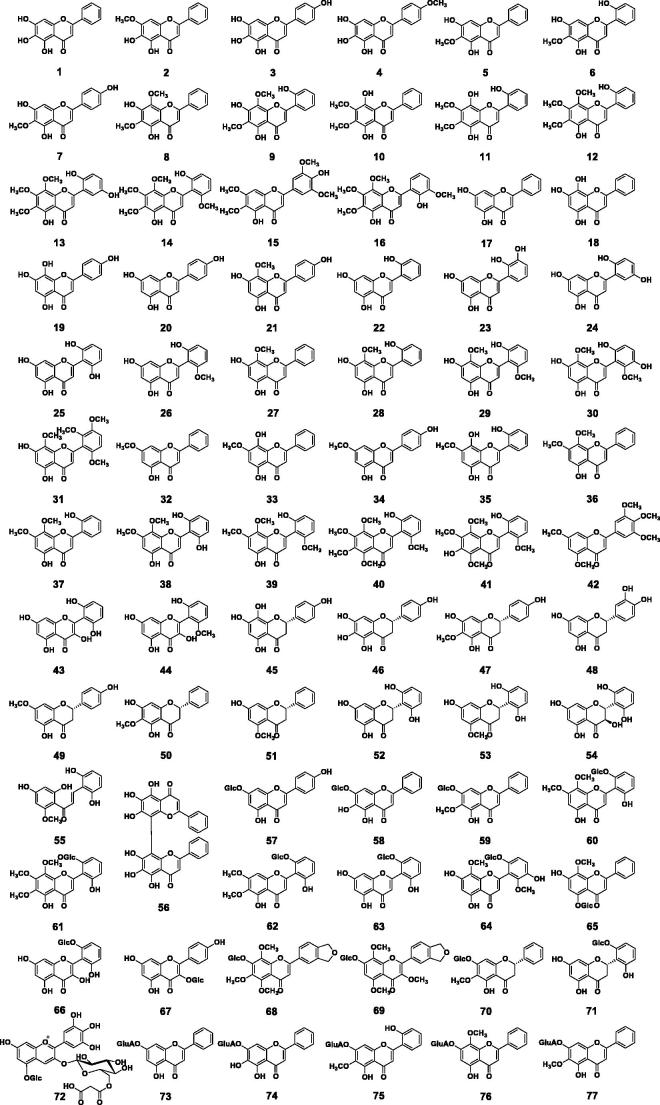

**Figure:**
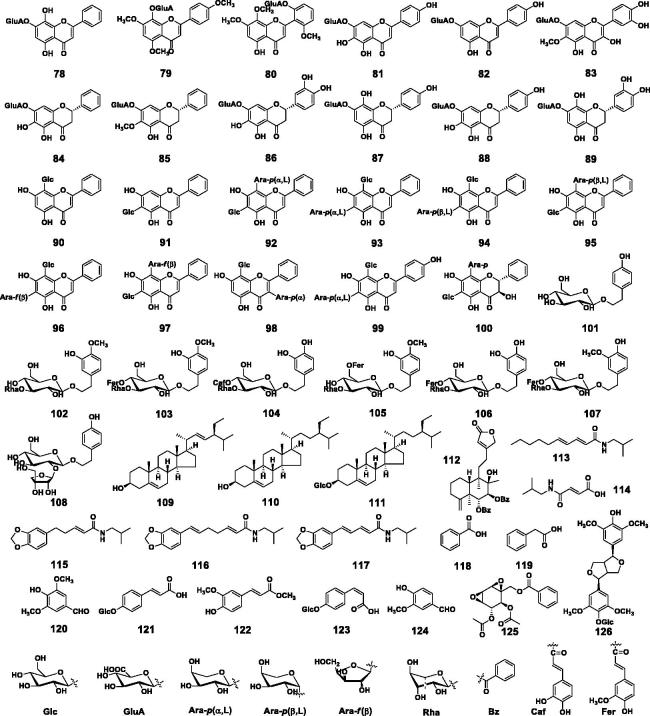

**Table 1.** Compounds **1**-**126** from *Scutellaria baicalensis*.

No.	Name	Molecular formula	Molecular weight	Plant part	References
**Free flavonoids**					
**1**	Baicalein (5,6,7-Trihydroxyflavone)	C_15_H_10_O_5_	270	Root; Hairy root	Popova et al. 1973;Ji et al. 2015; Zhou et al. 1997
**2**	5,6-Dihydroxy-7-methoxyflavone	C_16_H_12_O_5_	284	Root	Popova et al. 1973
**3**	Scutellarein (5,6,7,4'-Tetrahydroxyflavone)	C_15_H_10_O_6_	286	Root	Wang 2002
**4**	5,6,7-Trihydroxy-4'-methoxyflavone	C_16_H_12_O_6_	300	Root	Wang 2002
**5**	Oroxylin A (5,7-Dihydroxy-6-methoxyflavone)	C_16_H_12_O_5_	284	Root	Popova et al. 1973; Ji et al. 2015
**6**	Tenaxin II (5,7,2'-Trihydroxy-6-methoxyflavone)	C_16_H_12_O_6_	300	Root	Tomimori et al. 1983
**7**	5,7,4'-Trihydroxy-6-methoxyflavone	C_16_H_12_O_6_	300	Aerial part	Ma 2013
**8**	5,7-Dihydroxy-6,8-dimethoxyflavone	C_17_H_14_O_6_	314	Root	Wang 2002
**9**	5,7,2'-Trihydroxy-6,8-dimethoxyflavone	C_17_H_14_O_7_	330	Root	Wang 2002
**10**	5,8-Dihydroxy-6,7-dimethoxyflavone	C_17_H_14_O_6_	314	Root	Tomimori et al. 1982
**11**	5,8,2'-Trihydroxy-6,7-dimethoxyflavone	C_17_H_14_O_7_	330	Root	Takagi et al. 1980
**12**	Tenaxin I (5,2'-Dihydroxy-6,7,8-trimethoxyflavone)	C_18_H_16_O_7_	344	Root	Tomimori et al. 1983; Xu DY et al. 2011
**13**	5,2',5'-Trihydroxy-6,7,8-trimethoxyflavone	C_18_H_16_O_8_	360	Root	Tomimori et al. 1984a
**14**	Skullcapflavone II (5,6'-Dihydroxy-6,7,8,2'-tetramethoxyflavone)	C_19_H_18_O_8_	374	Root; Hairy root	Ishimaru et al. 1995; Zhou et al. 1997
**15**	5,4'-Dihydroxy-6,7,3',5'-tetramethoxyflavone	C_19_H_18_O_8_	374	Aerial part	Ma 2013
**16**	5,2'-Dihydroxy-6,7,8,3'-tetramethoxyflavone	C_19_H_18_O_8_	374	Hairy root	Nishikawa et al. 1999
**17**	Chrysin (5,7-Dihydroxyflavone)	C_15_H_10_O_4_	254	Root; Aerial part; Hairy root	Takagi et al.^1980^; Ma 2013; Zhou et al. 1997
**18**	Norwogonin (5,7,8-Trihydroxyflavone)	C_15_H_10_O_5_	270	Root	Popova et al. 1973; Tomimori et al. 1983
**19**	Isoscutellarein (5,7,8,4'-Tetrahydroxyflavone)	C_15_H_10_O_6_	286	Aerial part	Ma 2013
**20**	Apigenin (5,7,4'-Trihydroxyflavone)	C_15_H_10_O_5_	270	Root; Aerial part	Wang 2002; Ma 2013
**21**	4'-Hydroxywogonin(5,7,4'-Trihydroxy-8-methoxyflavone)	C_16_H_12_O_6_	300	Root	Tomimori. et al. 1982; Wang 2002
**22**	2'-Hydroxychrysin (5,7,2'-Trihydroxyflavone)	C_15_H_10_O_5_	270	Root	Tomimori et al. 1984a
**23**	5,7,2',3'-Tetrahydroxyflavone	C_15_H_10_O_6_	286	Root	Tomimori et al. 1984b
**24**	5,7,2',5'-Tetrahydroxyflavone	C_15_H_10_O_6_	286	Root	Zhang et al. 1994
**25**	5,7,2',6'-Tetrahydroxyflavone	C_15_H_10_O_6_	286	Root	Tomimori et al. 1982; Ishimaru et al. 1995
**26**	5,7,6'-Trihydroxy-2'-methoxyflavone	C_16_H_12_O_6_	300	Root	Tomimori et al. 1984a
**27**	Wogonin (5,7-Dihydroxy-8-methoxyflavone)	C_16_H_12_O_5_	284	Root; Aerial part; Hairy root	Popova et al. 1973; Ma 2013; Zhou et al. 1997
**28**	Scutevulin (5,7,2'-Trihydroxy-8-methoxyflavone)	C_16_H_12_O_6_	300	Root	Tomimori et al. 1984a
**29**	5,7,6'-Trihydroxy-8,2'-dimethoxyflavone	C_17_H_14_O_7_	330	Root	Tomimori et al. 1984a
**30**	Viscidulin III (5,7,3',6'-Tetrahydroxy-8,2'-dimethoxyflavone)	C_17_H_14_O_8_	346	Root	Tomimori et al. 1984a; Zhang et al. 1994
**31**	5,7-Dihydroxy-8,2',3',6'-tetramethoxyflavone	C_19_H_18_O_8_	374	Root	Long et al. 2015
**32**	7-Methoxychrysin (5-Hydroxy-7-methoxyflavone)	C_16_H_12_O_4_	268	Aerial part	Wang HW et al. 2016
**33**	5,8-Dihydroxy-7-methoxyflavone	C_16_H_12_O_5_	284	Root	Popova et al. 1973
**34**	Genkwanin (5,4'-Dihydroxy-7-methoxyflavone)	C_16_H_12_O_5_	284	Aerial part	Wang HW et al. 2016
**35**	5,8,2'-Trihydroxy-7-methoxyflavone	C_16_H_12_O_6_	300	Root	Takagi et al. 1980
**36**	7-*O*-Methylwogonin (5-Hydroxy-7,8-dimethoxyflavone)	C_17_H_14_O_5_	298	Root	Tomimori et al. 1983
**37**	Skullcapflavone I (5,2'-Dihydroxy-7,8-dimethoxyflavone)	C_17_H_14_O_6_	314	Root; Hairy root	Takido et al. 1979; Zhou et al. 1997
**38**	Viscidulin II (5,2',6'-Trihydroxy-7,8-dimethoxyflavone)	C_17_H_14_O_7_	330	Root	Tomimori et al. 1984a
**39**	Rivularin (5,6'-Dihydroxy-7,8,2'-trimethoxyflavone)	C_18_H_16_O_7_	344	Root; Hairy root	Zhang et al. 1994; Zhou et al. 1997
**40**	6'-Hydroxy-5,6,7,8,2'-pentamethoxyflavone	C_20_H_20_O_8_	388	Root	Wang 2002
**41**	6,6'-Dihydroxy-5,7,8,2'-tetramethoxyflavone	C_19_H_18_O_8_	374	Root	Wang 2002
**42**	5,7,3',4',5'-Pentamethoxyflavone	C_20_H_20_O_7_	372	Aerial part	Wang HW et al. 2016
**43**	Viscidulin І (5,7,2',6'-Tetrahydroxyflavonol)	C_15_H_10_O_7_	302	Root	Tomimori et al. 1984a;Ji et al. 2015
**44**	5,7,6'-Trihydroxy-2'-methoxyflavonol	C_16_H_12_O_7_	316	Root	Long et al. 2015
**45**	Isocarthamidin ((2*S*)-5,7,8,4'-Tetrahydroxyflavanone)	C_15_H_12_O_6_	288	Leaf; Root	Takido et al. 1976; Wang 2002
**46**	Carthamidin ((2*S*)-5,6,7,4'-Tetrahydroxyflavanone)	C_15_H_12_O_6_	288	Leaf; Root	Takido et al. 1976; Wang 2002
**47**	(2*S*)-5,7,4'-Trihydroxy-6-methoxyflavanone	C_16_H_14_O_6_	302	Root	Takagi et al^1980^
**48**	(+)-Eriodictyol ((2*S*)-5,7,3',4'-Tetrahydroxyflavanone)	C_15_H_12_O_6_	288	Root	Zhang et al. 1994
**49**	(2*S*)-5,4'-Dihydroxy-7-methoxyflavanone	C_16_H_14_O_5_	286	Aerial part	Wang HW et al. 2016
**50**	DihydrooroxylinA ((2*S*)-5,7-Dihydroxy-6-methoxyflavanone)	C_16_H_14_O_5_	286	Root	Takagi et al. 1980; Xu DY et al. 2011
**51**	(2*S*)-7-Hydroxy-5-methoxyflavanone	C_16_H_14_O_4_	270	Root	Xu DY et al. 2011
**52**	(2*S*)-5,7,2',6'-Tetrahydroxyflavanone	C_15_H_12_O_6_	288	Root	Kubo et al. 1981
**53**	(2*S*)-7,2',6'-Trihydroxy-5-methoxyflavanone	C_16_H_14_O_6_	302	Root	Tomimori et al. 1984a
**54**	(2*R*,3*R*)-3,5,7,2',6'-Pentahydroxyflavanone	C_15_H_12_O_7_	304	Root	Takagi et al. 1981b; Ji et al. 2015
**55**	2,6,2',4'-Tetrahydroxy-6'-methoxychalcone	C_16_H_14_O_6_	302	Root	Tomimori et al. 1984a
**56**	8,8''-Bibaicalein	C_30_H_18_O_10_	538	Root	Wang 2002
**Flavonoid glycosides**					
**57**	Apigenin 7-*O*-β-D-glucoside	C_21_H_20_O_10_	432	Aerial part	Ma 2013
**58**	Baicalein 7-*O*-β-D-glucoside	C_21_H_20_O_10_	432	Root; Aerial part	Tomimori et al. 1984a; Ma 2013
**59**	Oroxylin A 7-*O*-β-D-glucoside	C_22_H_22_O_10_	446	Aerial part; Root	Ma 2013; Ji et al. 2015
**60**	5,6'-Dihydroxy-7,8-dimethoxyflavone 2'-*O*-β-D-glucoside	C_23_H_24_O_12_	492	Root; Hairy root	Miyaichi et al. 1995; Zhou et al. 1997
**61**	5,6'-Dihydroxy-6,7,8-trimethoxyflavone 2'-*O*-β-D-glucoside	C_24_H_26_O_13_	522	Root	Ishimaru et al. 1995
**62**	5,6'-Dihydroxy-6,7-dimethoxyflavone 2'-*O*-β-D-glucoside	C_23_H_24_O_12_	492	Root; Hairy root	Ishimaru et al. 1995; Zhou et al. 1997
**63**	5,7,6'-Trihydroxyflavone 2'-*O*-β-D-glucoside	C_21_H_20_O_11_	448	Hairy root	Zhou et al. 1997
**64**	Viscidulin III 6'-*O*-β-D-glucoside	C_23_H_24_O_13_	508	Root; Hairy root	Zhang et al. 1994; Zhou et al. 1997
**65**	Wogonin 5-*O*-β-D-glucoside	C_22_H_22_O_10_	446	Root	Takagi et al. 1981b; Ji et al. 2015
**66**	3,5,7,6'-Tetrahydroxyflavone 2'-*O*-β-D-glucoside	C_21_H_20_O_12_	464	Root	Miyaichi et al. 1995
**67**	Kaempferol 3-*O*-β-D-glucoside	C_21_H_20_O_11_	448	Aerial part	Cha et al. 2006
**68**	5,6,8-Trimethoxy-3',4'-methylenedioxyflavone 7-*O*-β-D-glucoside	C_26_H_28_O_12_	532	Root	Lin et al. 2013
**69**	3,5,8-Trimethoxy-3',4'-methylenedioxyflavone 7-*O*-β-D-glucoside	C_26_H_28_O_12_	532	Root	Lin et al. 2013
**70**	(2*S*)-5-Hydroxy-6-methoxyflavanone 7-*O*-β-D-glucoside	C_22_H_24_O_10_	448	Root	Miyaichi et al. 1995
**71**	(2*S*)-5,7,6'-Trihydroxyflavanone 2'-*O*-β-D-glucoside	C_21_H_22_O_11_	450	Root	Ji et al. 2015
**72**	Delphinidin 3-*O*-(6-*O*-malonyl)-β-D-glucoside-5-*O*-β-D-glucoside	C_30_H_33_O_20_	713	Flower	Oszmianski et al. 2004
**73**	Chrysin 7-*O*-β-D-glucuronide	C_21_H_18_O_10_	430	Root; Aerial part; Root	Miyaichi et al. 1994; Ma 2013; Ji et al. 2015
**74**	Baicalin (5,6-Dihydroxyflavone 7-*O*-β-D-glucuronide)	C_21_H_18_O_11_	446	Root; Aerial part; Hairy root	Shibata et al. 1923; Ishimaru et al. 1995; Ma 2013; Zhou et al. 1997
**75**	5,2'-Dihydroxy-6-methoxyflavone 7-*O*-β-D-glucuronide	C_22_H_20_O_12_	476	Root	Miyaichi et al. 1994
**76**	Wogonoside (Wogonin 7-*O*-β-D-glucuronide)	C_22_H_20_O_11_	460	Root; Hairy root	Ishimaru et al. 1995;Ji et al. 2015; Zhou et al. 1997
**77**	Oroxyloside (Oroxylin A 7-*O*-β-D-glucuronide)	C_22_H_20_O_11_	460	Root	Zhang et al. 1997
**78**	Norwogonin 7-*O*-β-D-glucuronide (5,8-dihydroxyflavone 7-*O*-β-D-glucuronide)	C_21_H_18_O_11_	446	Root	Ji et al. 2015
**79**	Isoscutellarein 8-*O*-β-D-glucuronide	C_24_H_24_O_12_	504	Leaf	Nagai et al. 1989
**80**	5-Hydroxy-7,8,6'-trimethoxyflavone 2'-*O*-β-D-glucuronide	C_24_H_24_O_13_	520	Hairy root	Zhou et al. 1997
**81**	Scutellarin	C_21_H_18_O_12_	462	Root	Ji et al. 2015
**82**	Apigenin 7-*O*-β-D-glucuronide	C_21_H_18_O_11_	446	Aerial part	Cha et al. 2006
**83**	Patuletin 7-*O*-β-D-glucuronide (3,5,3',4'-Tetrahydroxy- 6-methoxyflavone 7-*O*-β-D-glucuronide)	C_22_H_20_O_14_	508	Root	Lin et al. 2013
**84**	Dihydrobaicalin ((2*S*)-5,6-Dihydroxyflavanone 7-*O*-β-D-glucuronide)	C_21_H_20_O_11_	448	Root	Tomimori et al. 1983
**85**	(2*S*)-5-Hydroxy-6-methoxyflavanone 7-*O*-β-D-glucuronide	C_22_H_22_O_11_	462	Root	Ji et al. 2015
**86**	(2*S*)-5,6,3',4'-Tetrahydroxyflavanone 7-*O*-β-D-glucuronide	C_21_H_20_O_13_	480	Aerial part	Liu et al. 2011
**87**	Isocarthamidin 7-*O*-β-D-glucuronide ((2*S*)-5,8,4'-Trihydroxyflavanone 7-*O*-β-D-glucuronide)	C_21_H_20_O_12_	464	Aerial part	Liu et al. 2011;Wang HW et al. 2016
**88**	Carthamidin 7-*O*-β-D-glucuronide (Dihydroscutellarein 7-*O*-β-D-glucuronide, Scutellarin B)	C_21_H_20_O_12_	464	Aerial part	Liu et al. 2011
**89**	(2*S*)-5,8,3',4'-Tetrahydroxyflavanone 7-*O*-β-D-glucuronide	C_21_H_20_O_13_	480	Aerial part	Liu et al. 2011
**90**	Chrysin 8-*C*-β-D-glucoside	C_21_H_20_O_9_	416	Root	Miyaich et al. 1994; Ji et al. 2015
**91**	Chrysin 6-*C*-β-D-glucoside	C_21_H_20_O_9_	416	Root	Miyaichi et al. 1994
**92**	Chrysin 6-*C*-β-D-glucoside-8-*C*-α-L-arabinopyranoside	C_26_H_28_O_13_	548	Root; Hairy root	Takagi et al. 1981a;Ji et al. 2015; Zhou et al. 1997
**93**	Chrysin 6-*C*-α-L-arabinopyranoside-8-*C*-β-D-glucoside	C_26_H_28_O_13_	548	Root; Hairy root	Takagi et al. 1981a; Ji et al. 2015; Zhou et al. 1997
**94**	Chrysin 6-*C*-β-L-arabinopyranoside-8-*C*-β-D-glucoside	C_26_H_28_O_13_	548	Root	Liu 2008
**95**	Chrysin 6-*C*-β-D-glucoside-8-*C*-β-L-arabinopyranoside	C_26_H_28_O_13_	548	Root	Liu 2008
**96**	Chrysin 6-*C*-β-arabinofuranoside-8-*C*-β-D-glucoside	C_26_H_28_O_13_	548	Root	Liu 2008
**97**	Chrysin 6-*C*-β-D-glucoside-8-*C*-β-arabinofuranoside	C_26_H_28_O_13_	548	Root	Liu 2008
**98**	Chrysin 3-*C*-α-arabinopyranoside-8-*C*-β-D-glucoside	C_26_H_28_O_13_	548	Root	Lin et al. 2013
**99**	Apigenin 6-*C*-α-L-arabinopyranoside-8-*C*-β-D-glucoside (isoschaftoside)	C_26_H_28_O_14_	564	Aerial part	Cha et al. 2006
**100**	(2*R*,3*R*)-Pinobankasin 6-*C*-glucoside-8-*C*-arabinopyranoside	C_26_H_30_O_14_	566	Root	Lin et al. 2013
**Phenylethanoid glycosides**					
**101**	Salidroside (4-Hydroxy-β-phenylethyl-β-D-glucoside)	C_14_H_20_O_7_	300	Hairy root	Zhou et al. 1997
**102**	Darendoside B	C_21_H_32_O_12_	476	Root	Miyaichi et al. 1995
**103**	Martynoside (2-(3-Hydroxy-4-methoxyphenyl) ethyl-1-*O*-α-L-rhamnosyl(1→3)-β-D-(4-feruloyl)-glucoside)	C_31_H_40_O_15_	652	Hairy root; Root	Zhou et al. 1997; Takagi et al. 1981b
**104**	Acteoside	C_29_H_36_O_15_	624	Hairy root; Root	Zhou et al. 1997; Miyaichi et al. 1994;Ji et al. 2015
**105**	Isomartynoside	C_31_H_40_O_15_	652	Root	Miyaichi et al. 1994
**106**	Leucosceptoside A	C_30_H_38_O_15_	638	Hairy root; Root	Zhou et al. 1997; Miyaichi et al. 1994
**107**	Cistanoside D	C_31_H_40_O_15_	652	Root	Ji et al. 2015
**108**	Darendoside A	C_19_H_28_O_11_	432	Root	Miyaichi et al. 1995
**Others**					
**109**	Stigmasterol	C_29_H_48_O	412	Root	Wang 2002
**110**	β-Sitosterol	C_29_H_50_O	414	Root	Xu DY et al. 2011
**111**	Daucosterin	C_35_H_60_O_6_	576	Root	Wang 2002
**112**	Scutebaicalin	C_34_H_38_O_7_	558	Aerial part	Hussein et al. 1996
**113**	Pellitorine	C_14_H_25_NO	223	Root	Xu et al. 2016
**114**	(*E*)-4-[(2-methylpropyl) amino]-4-*oxo*-2-butenoic acid	C_8_H_13_NO_3_	171	Root	Xu et al. 2016
**115**	Dihydropiperlonguminine	C_16_H_21_NO_3_	275	Root	Xu et al. 2016
**116**	Futoamide	C_18_H_23_NO_3_	301	Root	Xu et al. 2016
**117**	Piperlonguminine	C_16_H_19_NO_3_	273	Root	Xu et al. 2016
**118**	Benzoic acid	C_7_H_6_O_2_	122	Root	Xu DY et al. 2011
**119**	Phenyl acetic acid	C_8_H_8_O_2_	136	Root	Liu YX et al. 2009
**120**	Syringaldehyde	C_9_H_10_O_4_	182	Root	Xu et al. 2016
**121**	4-*O*-β-D-glucosyl-trans-*p*-coumaric acid	C_15_H_18_O_8_	326	Root	Liu YX et al. 2009
**122**	Ferulic acid methyl ester	C_11_H_12_O_4_	208	Root	Xu et al. 2016
**123**	4-*O*-β-D-glucosyl-cis-*p*-coumaric acid	C_15_H_18_O_8_	326	Root	Liu YX et al. 2009
**124**	Vanillin	C_8_H_8_O_3_	152	Root	Xu et al. 2016
**125**	(+)-Crotepoxide	C_18_H_18_O_8_	362	Root	Xu et al. 2016
**126**	(+)-Syringaresinol-*O*-β-D-glucoside	C_28_H_36_O_13_	580	Root	Miyaichi et al. 1994

### *Free flavonoids* (1–56)

A total of 56 free flavonoids have been isolated from *S. baicalensis.* They include 42 flavones (**1–42**), 2 flavonols (**43–44**), 9 flavanones (**45–53**), 1 flavonol (**54**), 1 chalcone (**55**), and 1 biflavonoid (**56**). The most abundant ones are baicalein (**1**), wogonin (**27**), and oroxylin A (**5**). Wogonin is the first free flavonoid isolated from *S. baicalensis*, and its structure was established in 1930 (Hattori 1930). Aside from the commonly seen C-5 and C-7 substituents, a number of *Scutellaria* flavonoids contain hydroxyl or methoxyl groups at C-6 and C-8, which are rare for plants. The regio-specific hydroxylation at C-6 and C-8 of flavones are catalyzed by two novel CYP450 enzymes (Zhao Q et al. 2018).

### *Flavonoid glycosides* (57-100)

Baicalin (**74**) is the most abundant compound of *S. baicalensis*. As the first pure compound reported from this plant, baicalin was originally reported by G. Bargellini in 1919 (Azimova and Vinogradova 2013), and its structure was established in 1923 (Shibata et al. 1923). Today, 44 flavonoid glycosides have been reported from *S. baicalensis*. They can be classified into *O*-glucosides (**57–72**), *O*-glucuronides (**73–89**), and *C*-glycosides (**90–100**).

For most of the *O*-glucosides, the glucosyl residues are substituted at 7-OH or 2′-OH. Wogonin 5-*O*-β-d-glucoside (**65**), kaempferol 3-*O*-β-d-glucoside (**67**), and **72** are exceptions. Compound **72** is an acylated anthocyanin containing two glucosyl residues at C-3 and C-5, and contributes to the blue (or purple) color of the flowers (Oszmiański et al. 2004).

While glucuronides are not as prevalent as glucosides in plant secondary metabolites, *S. baicalensis* contains at least 17 *O*-glucuronides. Baicalin (**74**) and wogonoside (**76**) are the most abundant ones. For majority of these compounds, the glucuronyl group is linked to 7-OH, except for **79** (8-OH) and **80** (2′-OH).

The first two *C*-glycosides were reported from *S. baicalensis* in 1994 (Miyaichi and Tomimori 1994). Up to now, 11 *C*-glycosides have been isolated from this plant. Most of them are glycosides of chrysin, though it is not the most abundant free flavonoid in *S. baicalensis*. Aside from two mono-*C*-glucosides, majority of the other compounds are 6,8-di-*C*-glycosides, containing one glucosyl residue and one arabinosyl residue. Interestingly, the arabinosyl residue in these compounds occurred as both furano- and pyrano- forms, and in different configurations (α-l, β-l). Their structures were mainly determined by NMR spectroscopic analysis. Unlike *O*-glycosides, the sugar residues are not easily hydrolyzed to identify their forms and stereo-configurations. Structures for some *C*-glycosides need to be further confirmed.

### *Phenylethanoid glycosides* (101–108)

A total of nine phenylethanoid glycosides have been reported from *S. baicalensis.* The aglycones are usually conjugated with a glucosyl group, which are further substituted with a rhamnosyl residue (Rha), or acylated with a caffeoyl (Caf) or feruloyl (Fer) group.

### *Other small molecules* (109–126)

The other types of small molecules isolated from *S. baicalensis* include three steroids (**109–111**), one diterpene (**112**), five amides (**113**-**117**), and nine phenolic compounds (**118–126**). The amides are conjugates of isobutyl amine and organic acids, and were isolated from a water extract by Xu et al. (2016).

### Polysaccharides

Olennikov and colleagues isolated five polysaccharides from the aerial part of S. *baicalensis*. They were named as WSPS′-1, WSPS′-2, WSPS′-3, WSPS′-4, and WSPS′-5. Among them, WSPS′-1, WSPS′-2, and WSPS′-3 are composed of arabinose, galactose and glucose, whereas WSPS′-4 and WSPS′-5 are composed of glucose (Olennikov et al. 2008a, 2008b). The same research group also obtained a homopolysaccharide SbRP-1′′ from the roots of *S. baicalensis*. SbRP-1′′ is a slightly branched glucan. The main chain is composed of α-(1 → 4)-glucopyranose units, 8.3% of which are substituted with an α-glucopyranose unit at C-6 (Olennikov et al. 2011).

### Qualitative and quantitative analyses

With the rapid development of mass spectrometry techniques, liquid chromatography coupled with mass spectrometry (LC/MS) has been widely used to characterize the chemical constituents in herbal extracts. A number of reports are available on chemical analysis of *S. baicalensis* to characterize tens of compounds within 1 h (Han et al. 2007; Liu GZ et al. 2009). Wang et al. (2013) depleted high-abundance flavonoids from an ethanol extract of *S. baicalensis*, and characterized 117 low-abundance compounds by LC/MS. Recently, our group established a targeted post-acquisition data processing strategy, key ion filtering (KIF), and tentatively characterized 132 compounds in Huang-Qin by ultra-high performance liquid chromatography coupled with hybrid quadrupole orbitrap mass spectrometry analysis (UHPLC/Orbitrap-MS) (Qiao et al. 2016). Among these compounds, 59 were reported in this herb for the first time.

**Figure 3.**
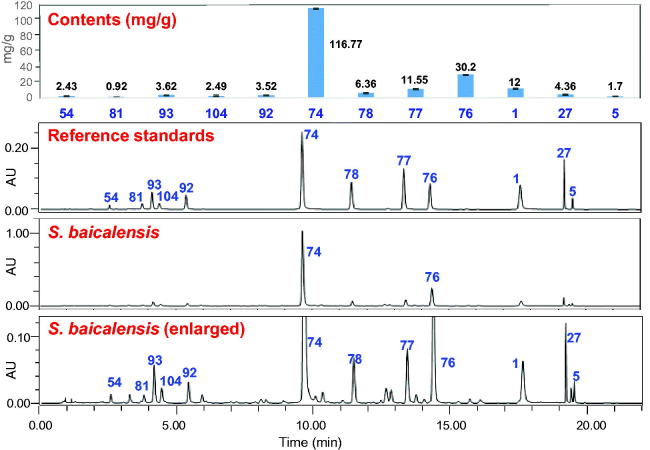
UPLC/UV chromatograms (275 nm) for quantitative analysis of 12 major compounds in *Scutellaria baicalensis* (Huang-Qin crude drugs). **1**, baicalein; **5**, oroxylin A; **27**, wogonin; **54**, (2*R*,3*R*)-3,5,7,2′,6′-pentahydroxyflavanone; **74**, baicalin; **76**, wogonoside; **77**, oroxylinA 7-*O*-β-D-glucuronoside; **78**, norwogonin 7-*O*-β-D-glucuronoside; **81**, scutellarin; **92**, chrysin 6-*C*-β-D-glucoside-8-*C*-α-L-arabinopyranoside; **93**, chrysin 6-*C*-α-L-arabinopyranoside-8-*C*-β-D-glucoside; **104**, acteoside (Adapted from Ji et al. 2015).

The contents of bioactive compounds are critically important for quality control of herbal medicines. Chinese Pharmacopoeia requires the content of baicalin in Huang-Qin should be no less than 9% (Chinese Pharmacopoeia Commission 2015). A number of HPLC methods have been developed to determine the contents of baicalin and other bioactive compounds in Huang-Qin (Xie et al. 2002; Zgórka and Hajnos 2003; Horvath et al. 2005; Islam et al. 2012). We developed a simple and rapid UPLC/UV method, and simultaneously determined the contents of 12 compounds in *S. baicalensis* within 20 min (Ji et al. 2015). Contents of these 12 compounds in 27 batches of Huang-Qin accounted for around 19.6% of dry weight of the herbal materials (Figure 3).

## Pharmacological activities of extracts and major compounds

**Figure 4.**
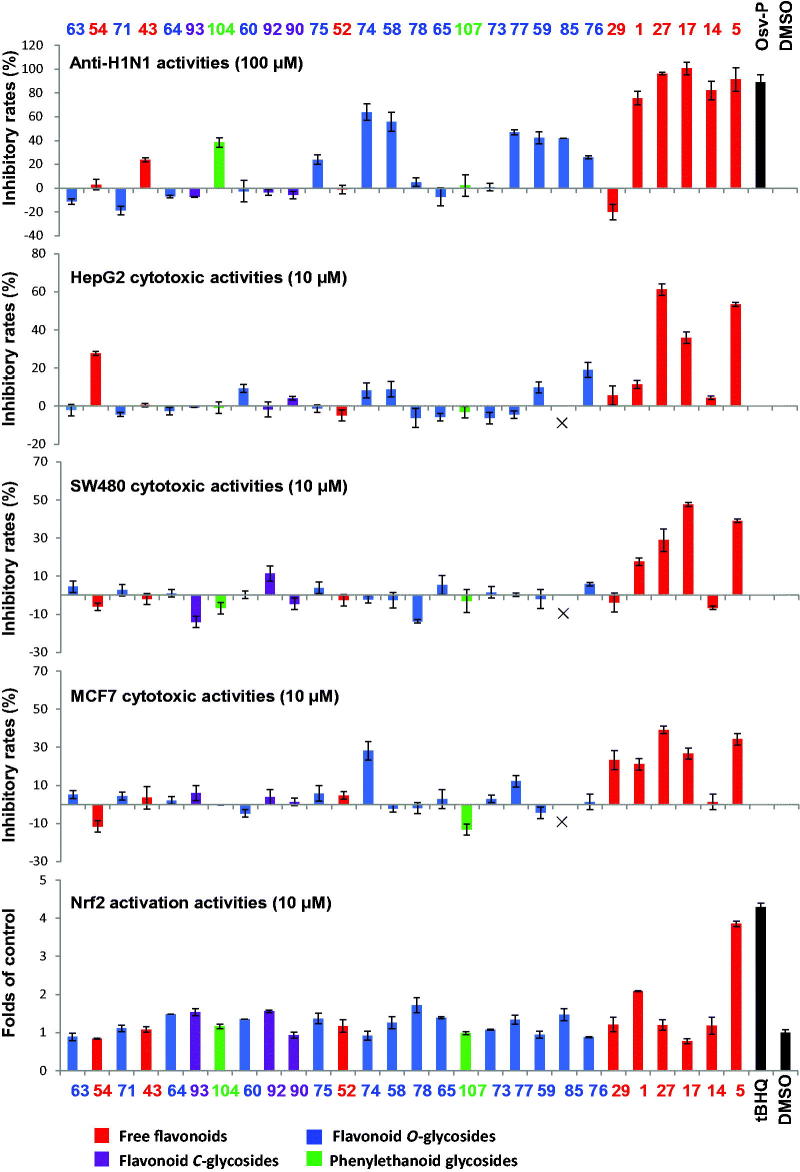
Screening of 28 compounds from *Scutellaria baicalensis* for their anti-H1N1 viral, cytotoxic, and Nrf2 activation activities. For compounds identification, see Table 1 (Adapted from Ji et al. 2015).

In China, Huang-Qin is widely used for the treatment of influenza, pneumonia, dysentery, and cancer. A large number of investigations have been reported on the pharmacological activities of different extracts of *S. baicalensis* (including water extract, methanol extract, and ethanol extract) and its major compounds such as baicalin, baicalein, and wogonin. These results were reported by different research groups, the investigations were conducted using different experimental models, and thus the results were difficult to be compared or summarized. Recently, our group isolated 28 compounds from this herb, and evaluated their anti-H1N1 viral, cytotoxic, and Nrf2 activation activities (Ji et al. 2015). The results indicated that free flavones were more potent than the other types as anti-influenza, cytotoxic, and antioxidative compounds of *S. baicalensis* (Figure 4). They may be key players in the clinical therapeutic effects of Huang-Qin.

In this section, we summarize literature reports on the anti-tumor, anti-viral, anti-microbial, anti-inflammatory, antioxidative, neuroprotective, and hepatoprotective activities of extracts and compounds of *S. baicalensis*, as well as their effects on cardiovascular and cerebrovascular diseases, and bones.

### Anti-tumor activities

*Scutellaria baicalensis* extracts and compounds have been reported to show a wide spectrum of anti-tumor activities, both *in vitro* and *in vivo* (Table 2). These activities involve liver cancer, gastric cancer, lung cancer, breast cancer, prostate cancer, bladder cancer, brain cancer, squamous cell carcinoma, mucoepidermoid carcinoma, colorectal cancer, gallbladder carcinoma, oral cancer, leukemia, lymphoma, and myeloma.

**Table 2.** The anti-tumor activities of *Scutellaria baicalensis* and its compounds.

Drugs	Dose	Model	Efficacy	Mechanism	References
Water extract	20–800 μg/mL	HepG2 liver cancer cells	IC_50_, 360 μg/mL	G2/M phase arrest	Ye et al. 2009
Water extract	10–500 μg/mL	A549 lung cancer cells	Inhibited cell motility at >250 μg/mL	Inhibition of MMP-2 activity and cell motility	Park et al. 2011
Water extract	40–800 μg/mL 200 mg/kg five times per week for 7 weeks i.g.	Prostate cancer cell lines (LNCaP, PC-3); PC-3 prostate cancer xenograft NCR nude mice	IC_50_, 100–150 μg/mL; Tumor inhibition rate, 50%	Inhibition of COX-2 activity; G1 and G2/M phase arrest	Ye F et al. 2007
Water extract	1.5–1500 μg/mL 75 mg/kg five times/week for 7 weeks, p.o.	Head and neck squamous cell carcinoma (SCC-25, KB); KB HNSCC xenograft female NCR/NU nude mice	IC_50_, 150 μg/mL; Tumor inhibition rate, 66%	Inhibition of PGE2 synthesis via suppression of COX-2 expression	Zhang et al. 2003
Methanol extract	400 μg/mL	HepG2 liver cancer cells	Inhibition rate, 44.4%	Regulation of MMP-2 and FOXM1 activities	Park et al. 2014
Ethanol extract	7.8–250 μg/mL	Lung cancer cell lines (A549, SK-LU-1, SK-MES-1)	IC_50_, 57.2–102.1 μg/mL	S and G0/G1 phase arrest; increased expression of p53 and Bax	Gao et al. 2011
Ethanol extract	0–100 μg/mL	Cell lines derived from primary and recurrent brain tumors from patients	IC_50_, 50–100 μg/mL; Increasing inhibitory effect with anti-tumor drug BCNU		Scheck et al 2006
Total free flavonoid extract	25 and 100 mg/kg for 30 d, p.o.	A549 human lung cancer xenograft female BALB/c nude mice	Tumor inhibition rate, 25.5%	S phase arrest; inhibition of DNA synthesis	Wang Y et al. 2016
Fraction (containing baicalein and wogonin)	1–100 μg/mL	MCF-7 breast cancer cells	Inhibition rate, 81.6% at 100 μg/mL	S-phase and G2/M-phase arrest; increasing cell apoptosis	Wang et al. 2010
Extracts	10^-3^–10^3^ μg/mL	Myeloid leukemia cells (HL-60, NB-4, THP-1, U937), lymphocytic leukemia cells (Blin-1, Nalm-6), lymphoma cell lines (Daudi, Raji, Ramos, NCEB1), myeloma cell lines (NCIH929, U266)	IC_50_, 4.57–12.3 μg/mL	Modulation of the Bcl family of genes and mitochondrial damage	Kumagai et al. 2007
Baicalin	25–800 μM	Prostate cancer cell lines (DU145, PC-3, LNCaPFGC, CA-HPV-10)	Cytotoxic for several human prostatic cancer cell lines; IC_50_, 150 μM for DU145 cells	Induction of apoptosis	Chan et al. 2000
Baicalin	3.2–320 μg/mL; 50, 100, 200 mg/kg five times/week for 2 weeks, i.p.	Mc3 mucoepidermoid carcinoma cells; Mc3 mucoepidermoid carcinoma xenograft BALB/c nude mice	IC_50_, 40 μg/mL; Tumor inhibition rate, about 50% at high dose	G0/G1 and G2/M phase arrest; decreasing the mitochondrial membrane potential	Xu XF et al. 2011
Baicalein	20 mg/kg/d for 2 weeks p.o.	LnCaP 35 prostate cancer xenograft BALB/c nude mice	Tumor inhibition rate, 55%	Reduce expression of the androgen receptor and androgen-regulated genes	Bonham et al. 2005
Baicalein	0–100 μM; 0.8 mg/mouse for 9 times for 21 d, i.p.	5637 bladder tumor cells; MB49 bladder cancer xenograft female C57BL/6 mice	G1 phase and S phase arrest at <100 μM	G1 and S phase arrest; inhibition of AKT, GSK3β, ERK, p38, and p65NF-κB, p65NF-κB	Wu et al. 2013
Baicalein	50–400 μM	Head and neck squamous cell carcinoma (SCC-25, KB)	IC_50_, 75 μM	Inhibition of PGE2 synthesis via suppression of COX-2 expression	Zhang et al. 2003
Baicalein	0–60 μM; 15 and 50 mg/kg/day for 1 week, i.g.	SGC-7901 gastric cancer cells; SGC-7901 gastric cancer xenograft BALB/c nude mice	IC_50_, about 30 μM; Tumor inhibition rate, about 50% at high dose	S phase arrest; Inducing apoptosis through the mitochondrial pathway	Mu et al. 2016
Baicalein	20–100 μM; 30 mg/kg/2 d for 4 weeks, i.p.	HCT-116 human colorectal cancer cells; HCT-116 human colorectal cancer xenograft nude mice	IC_50_, 40.1 μM; Tumor inhibition rate, about 50%	S phase arrest and pro-apoptotic effects; inducing the activation of caspase 3 and 9	Wang CZ et al. 2015
Baicalein	7–56 μM	HSC-3 oral cancer cells	IC_50_, ∼50 μM	G1 phase arrest; enhancing the degradation of cyclin D1 and activating AhR to decrease Rb phosphorylation	Cheng YH et al. 2012
Wogonin	1–100 μM	GBC-SD human gallbladder carcinoma cells	IC_50_, ∼50 μM	Inhibiting cell mobility and invasion by upregulating the metastasis suppressor maspin	Dong et al. 2011
Wogonin	0.1, 1, 10 mg/kg	T47D or MDA-MB-231 breat cancer xenograft female nude BALB/c mice	Tumor inhibition rate, 88% at high dose	Downregulation of the Akt-dependent canonical Wnt signaling pathway and p27^kip^ pathway; downregulation of ERa and c-ErbB2	Chung et al. 2008
Baicalin, wogonin, baicalein		Leukemia cell lines (CCRF-CEM, K562, P3HR-1, Raji, U937)	IC_50_, 10.6-20.5 μg/mL	Cellular DNA fragmentizing and G0/G1 phase arrest	Shieh et al. 2006
Wogonin, water extract	10–100 μM; 0.5–3 mg/mL	HL-60 leukemia cells	IC_50_, ∼50 μM (wogonin); ∼1 mg/mL (extracts)	Induction of Bax/Bcl-2 apoptosis and telomerase inhibition suppression of c-myc	Huang et al. 2010
Wogonoside	80 mg/kg/2 d for 14 d, i.p.	U937 acute myeloid leukemia xenograft BALB/c nude mice	Tumor inhibition rate, 41%	Cell cycle arrest and differentiation by affecting expression and subcellular localization of PLSCR1	Chen et al. 2013

The extracts of *S. baicalensis* could inhibit the proliferation of human myeloma, lung cancer, liver cancer, and prostate cancer cells *in vitro*, and suppress tumor growth in bladder, prostate, lung and head/neck squamous xenograft tumor models. In the head/neck squamous cell carcinoma (HNSCC) murine model, oral administration of a water extract (75 mg/kg, 5 times/week for 7 weeks) led to 66% reduction of xenograft tumor (Zhang et al. 2003). The anti-cancer activities of *S. baicalensis* could be related with its inhibitory effects on PGE_2_ (prostaglandin E2) production via suppression of COX-2 (cyclooxygenase-2) expression and arachidonic acid release from cell membranes. The total free flavonoid extract (100 mg/kg for 30 d, p.o.) could also significantly reduce tumor size by 25.5% in A549 human lung cancer xenografted mice, via induction of growth arrest in S phase and inhibition of DNA synthesis (Wang Y et al. 2016).

The main flavones baicalin (**74**), baicalein (**1**), wogonin (**27**), and wogonoside (**76**) are the major bioactive constituents responsible for the anti-tumor activities, with IC_50_ values of 10–50 μM against most tested cancer cell lines *in vitro* (Chan et al. 2000; Chen et al. 2008; Wu et al. 2013). These flavones could scavenge oxidative radicals, attenuate NF-κB (nuclear factor-κB) activity, suppress COX-2 gene expression, and regulate cell cycle (Li-Weber 2009). Baicalin (200 mg/kg, 5 times/week for 2 weeks, i.p.) could inhibit mucoepidermoid carcinoma Mc3 cell growth by 50% in the xenograft murine model (Xu XF et al. 2011). It could suppress cell cycle progression and induce cell apoptosis through decreasing the mitochondrial membrane potential.

Baicalein (20 mg/kg, 5 d/week for 21 d, i.p.) could inhibit MDA468 breast cancer xenografts by 40%, the effect of which was comparable to that of the positive drug cisplatin (5 mg/kg). It could upregulate DDIT4 (DNA-damage-inducible transcript 4) expression, which mediated the inhibition of mTOR (mammalian target of rapamycin) (Wang YJ et al. 2015). In another prostate cancer xenograft murine model, baicalein (20 mg/kg for 14 d, p.o.) reduced tumor size by 55%, via reduction of expression of the androgen receptor and androgen-regulated genes (Bonham et al. 2005).

Wogonin (10 mg/kg for 4 weeks, p.o.) could inhibit tumor growth of T47D and MDAMB-231 breast cancer xenografts by up to 88% without significant toxicity in athymic nude mice (Chung et al. 2008). The mechanism could be downregulation of the Akt-dependent canonical Wnt signaling pathway and p27^kip^ pathway. Wogonin could also act as CDK (cyclin-dependent kinase) inhibitors to potentiate the activities of anti-tumor drugs, such as the Bcl-2 (B-cell lymphoma 2) family inhibitor ABT-263. The combination of wogonin (50 mg/kg for 10 d, i.p.) and ABT-263 remarkably promoted tumor regression in human T-cell leukemia xenografted mice, but wogonin did not exhibit significant effects when used alone (Polier et al. 2015).

Wogonoside exerted anti-proliferative properties, suppressing tumor growth by 41% and prolonging survival durations up to 2.3-fold, in a U937 leucocythemia xenograft murine model (80 mg/kg/2 d for 14 d, i.p.) (Chen et al. 2013). The anti-tumor effect of wogonoside was related to cell cycle arrest and differentiation via inhibition of PLSCR1 (phospholipid scramblase 1) expression and regulation of subcellular localization in the nucleus.

PHY906 is an herbal preparation derived from the traditional Chinese medicine formula Huang-Qin Decoction, a four-herb formula with Huang-Qin as the key component (Ye M et al. 2007). PHY906 could enhance the anti-tumor activities of sorafenib against HepG2 tumor both *in vivo* and *in vitro*. Among the four component herbs, *S. baicalensis* played an important role in increasing tumor apoptosis by multiple mechanisms targeting on the inflammatory state of microenvironment of tumor tissue (Lam et al. 2015). PHY906 could also decrease gastrointestinal toxicity caused by the chemotherapeutic drug irinotecan. In a murine MCA-38 allograft model, PHY906 remarkably increased the anti-tumor activities of irinotecan and decreased weight loss (Lam et al. 2010).

### Anti-viral activities

*Scutellaria baicalensis* extracts and compounds exerted broad-spectrum anti-viral activities against HIV, influenza virus, DENV, HBV, and HTLV-I.

The extracts of *S. baicalensis* could inhibit HIV on H9 cells (IC_50_, 0.6–4.74 μg/mL), DENV on Vero cells (86.59–95.19 μg/mL), and H1N1 and seasonal influenza A viruses on MDCK cells (14.16–41.49 μg/mL) (Zhang et al. 1991; Hour et al. 2013; Zandi et al. 2013). These investigations were conducted on cell models.

Baicalein (480 mg/kg for 4 d, p.o.) showed significant effects in preventing death, prolonging survival time, inhibiting lung consolidation, and reducing the viral titers in the lung in BALB/c mice infected with the influenza A/FM1/1/47 (H1N1) virus. The effects were comparable to lamivudine. The mechanism could be inhibition of neuraminidase activity and modulation of the immune system (Xu et al. 2010). The combination of baicalein (400 mg/kg for 5 d, p.o.) and ribavirin (50 mg/kg) provided a higher survival rate and lower body weight loss than either treatment alone in ICR mice infected with H1N1 virus (protection rates, 100% vs 20% and 50%) (Chen et al. 2011).

Wogonin could suppress HBV antigen secretion with an IC_50_ of 4 μg/mL for both HBsAg and HBeAg in the human HBV-transfected liver cell line HepG2.2.15, and was more potent than lamivudine. *In vivo*, wogonin (i.v. for 10 d) could reduce plasma duck hepatitis B virus (DHBV) DNA level in the liver of DHBV-infected ducks with an ED_50_ of 5 mg/kg, via inhibition of DHBV DNA polymerase and thus reducing the relaxed circular and linear forms of DHBV DNA (Guo et al. 2007).

5,7,4′-Trihydroxy-8-methoxyflavone (**21**, 50 μM) could remarkably inhibit influenza virus A/PR/8/34 (APR8) by reducing the replication of APR8 in MDCK cells, through inhibition of the fusion of the virus with endosome/lysosome membrane at early stage and inhibition of the budding of the progeny virus from the cell surface (Nagai et al. 1995).

Furthermore, baicalein, baicalin, and wogonin could also inhibit other types of viruses, including HIV, herpes simplex virus-1 (HSV-1), Moloney murine leukemia virus, and Rous-associated virus type 2 (Baylor et al. 1992; Li et al. 1993, 2000a; Kitamura et al. 1998; Huang et al. 2000; Wang et al. 2004; Guo et al. 2007; Błach-Olszewska et al. 2008; Nayak et al. 2014). Recently, Lin et al. (2016) reported that *S. baicalensis* could be used to treat severe HFMD (Hand, Foot, and Mouth Disease) in patients aged >1 year, rapidly relieving fever, attenuating oral lesions and rashes, and improving nervous system involvement. This result was derived from a multi-center and retrospective analysis (Lin et al. 2016). It is reasonable to assume that *S. baicalensis* and its compounds possess a common, non-specific anti-viral mechanism, based on its inhibitory effects on different types of viruses.

### Anti-microbial activities

*Scutellaria baicalensis* and its major compounds possess remarkable anti-microbial activities. The water extract of *S. baicalensis* could inhibit a wide spectrum of oral bacteria (MIC, 15.7–62.5 mg/mL; MBC, 20–125 mg/mL), including *Streptococcus sanguis* II, *S. salivarius*, *Actinomyces viscosus*, *A. naeslundii*, *A. odontolyticus*, two strains of *Capnocytophaga*, *Bacteroides melaninogenicus* ss *intermedius*, *B. gingivalis*, *Fusobacterium nucleatum*, and *Actinobacillus actinomycetemcomitans* (Tsao et al. 1982). It could also inhibit the growth of *Candida albicans* by 90% at 2.5 mg/mL (Wong and Tsang 2009).

Baicalin (100 mg/kg, p.o.) could protect mice from staphylococcal pneumonia caused by *Staphylococcus aureus*, reducing mortality from 80% to 28% and protecting the lung from accumulation of cellular infiltrates (Qiu et al. 2012). This activity is associated with inhibition of the cytolytic activity of α-hemolysin, which is a self-assembling and channel-forming toxin secreted by *S. aureus*. Baicalein also showed potent synergistic effect with penicillin G/amoxicillin against 20 clinical penicillinase-producing *S. aureus* strains. Baicalin at 32 μg/mL could enhance the bacteriostatic effects, and decrease the MIC_50_ values of penicillin and amoxicillin from 32–64 to 0.5–2 μg/mL (Qian et al. 2015).

Viscidulin (**43**, 5,7,2′,6′-tetrahydroxyflavanonol, 40 mg/kg for one time, i.v.) could protect mice against a lethal challenge with heat-killed *Escherichia coli* 35218, increasing the survival rate from 0% to 60% via neutralization of LPS (lipopolysaccharide) and reduction of proinflammatory cytokines (Fu et al. 2008).

### Anti-inflammatory activities

An extract of *S. baicalensis* (750 mg/kg for 10 d, p.o.) showed potent anti-inflammatory activities in the zymosan-induced mice air-pouch, reducing NO production from 30 to 5 μM, through the down-regulation of IKKαβ (IκB kinase αβ) and NF-κB activation via suppression of c-Raf-1/MEK1/2 and MAPK phosphorylation (Kim et al. 2009). The flavonoids extract (100 μg/mL) also exhibited significant anti-inflammatory activities through inhibiting the NF-κB signaling pathway via the MAPK (mitogen-activated protein kinase) signaling pathway in RAW264.7 cells (Hong et al. 2013).

Baicalein (50–100 μM) showed anti-inflammatory effects in double-stranded RNA (dsRNA)-induced macrophages by inhibiting NO, cytokines, chemokines, and growth factors via the endoplasmic reticulum stress-CHOP/STAT pathway (Kim et al. 2018). Baicalin (100 mg/kg for 7 d, i.p.) could relieve ankle swelling, and protect the joint against inflammatory destruction in a murine adjuvant-induced arthritis model, by inhibiting splenic Th17 cell expansion and IL-17 (interleukin 17A)-mediated inflammation in synoviocytes (Yang X et al. 2013). Furthermore, baicalin (200 mg/kg for 7 d, p.o.) could alleviate LPS-induced liver inflammation in chicks, reducing the cloacal temperature from 41.5 to 40.3 °C and inhibiting NO production from 105 to 40 μM, via suppression of TLR4 (Toll-like receptor 4)-mediated NF-κB pathway (Cheng et al. 2017). Baicalin could also decrease inflammation by selective binding to chemokine ligands on CD4 and other leukocytes (Li et al. 2000b). Wogonoside (50 μM) could decrease the production of inflammatory mediators NO and PGE2, and inhibit the release of pro-inflammatory cytokines including TNF-α (tumor necrosis factor α) and IL-6 in LPS-induced RAW264.7 cells (Yang YZ et al. 2013). Wogonin treatment also regulated the production of inflammatory cytokines in mice with streptozotocin-induced vascular inflammation (Wang J et al. 2017).

### Antioxidative activities

The extract of *S. baicalensis* (1 mg/mL) could protect cardiomyocytes *in vitro* from moderate hypoxia, ischemia/reperfusion, and antimycin A exposure, decreasing cell death from 47–49% to 23–26% by scavenging ROS (reactive oxygen species) (Shao et al. 1999).

Baicalein, baicalin, and wogonin showed potent antioxidative activities by scavenging ONOO^-^ and inhibiting ONOO^-^-mediated nitrotyrosine formation in endothelial cells with IC_50_ values of 0.71–6.70 μM, the activity of which was comparable to penicillamine (3.75 μM) (Kim et al. 2005). Baicalein and baicalin exhibited antioxidant activities against hydroxyl radical, DPPH (2,2-diphenyl-1-picrylhydrazyl) radical, and alkyl radical, with IC_50_ values of 10–32 μM (Gao et al. 1999). Baicalein (50 μM) also exhibited antioxidative activity in ischemia/reperfusion cardiomyocyte model, decreasing subsequent cell death from 52.3% to 29.4% (Shao et al. 2002).

### Neuroprotective activities

The extract of *S. baicalensis* (200 mg/kg for 40 or 32 d, p.o.) could improve rat act in the Morris water assay, reducing search error to about 50% in the chronic cerebral hypoperfusion and the LPS infusion models (Hwang et al. 2011). Treatment with the extract attenuated the neuroinflammatory responses and reduced the spatial memory impairments, via mitigating alterations of hippocampal MAPK signaling (Table 3). The extact could also protect animals from global cerebral ischemia and MPTP (1-methyl-4-phenyl-1,2,3,6-tetrahydropyridine)-induced Parkinson's disease, and protect cortical and neuronal cells from glutamate, NMDA (*N*-methyl-D-aspartic acid), and H_2_O_2_ induced toxicity *in vitro* (Yang et al. 2014; Cao et al. 2016; Li et al. 2016).

**Table 3.** The neuroprotective activities of *Scutellaria baicalensis* and its compounds.

Drugs	Dose	Experimental model	Efficacy	Mechanism	References
Methanol extract	0.1–1mg/kg for 7 d, i.p.	Global cerebral ischemia Wistar rats	89.6% protection of neuronal cell density	Inhibiting proinflammatory events (TNF-αand NO) and oxidative stress	Kim et al. 2001
Ethanol extract	1–100 μg/mL	Glutamate- and NMDA-induced primary rat cortical cell excitotoxicity	Protection rate, 90–95% at high dose	Inhibition of NMDA receptor function by interacting with the glycine binding site of the NMDA receptor	Yang J et al. 2014
Water extract	50 μg/mL	H_2_O_2_-induced neuronal HT-22 cell injury	Protection rate, 80–90%	Increasing the Bcl-2 level and decreasing the Bax level	Choi et al. 2002
Stem and leave extracts	18–76 μg/mL	H_2_O_2_-induced PC12 cell injury	Protection rate, >90% at high dose	Elevating the activity of SOD and Na^+^-K^+^-ATPase and lowering the MDA level and LDH release	Shang et al. 2006
Stem-leaf total flavonoids	50, 100 mg/kg for 60 d, i.p.	Chronic cerebral ischemia-induced vascular dementia of SD rats	Improving spatial learning and memory at high dose	Regulating kinases-triggered phosphorylation and PP2A-catalyzed dephosphorylation	Cao et al. 2016
Stem-leaf total flavonoids	5 mg/kg for 5 d, i.v.	MPTP-induced Parkinson's disease in C57BL/6J mice	Higher Hanging test scores; improving the behaviors and the numbers of dopaminergic neurons in the substantia nigra	Reduction in serum malondialdehyde and inhibition of oxidation, alleviating the damage of oxygen free radicals to dopaminergic neurons	Li et al. 2016
Wogonin	1–300 μg/mL	Glutamate, *N*-methyl-*D*-aspartic acid, H_2_O_2_, xanthine/xanthine oxidase, BSO, Fe^2+^ and L-ascorbic acid induced primary rarat cortical cell toxicity	EC_50_, 6.8–143.3 μg/mL	Radical scavenging	Cho and Lee 2004
Baicalein	1–5 μM	LPS-induced primary rat embryo midbrain neuron-glia damage	Attenuating LPS-induced decrease in dopamine uptake and loss of TH-immunoreactive neurons	Inhibition of LPS-induced production of NO and free radicals from microglia	Li et al. 2005
Baicalein	200 mg/kg for a week, i.p.	MPTP-induced Parkinson's disease in C57BL/6J mice	Improving the abnormal behavior	Increasing the levels of DA and 5-HT in the striatum and the counts of dopaminergic neurons, inhibiting oxidative stress and the astroglia response	Cheng et al. 2008
Baicalein	200 mg/kg for 3 weeks, i.p.	6-Hydroxydopamin-induced experimental parkinsonism SD rats	Decreasing the burst frequency and amplitude of muscle activity to 13.43% and 35.18%	Increasing the number of dopaminergic neurons related with anti-apoptotic, pro-differentiation and anti-inflammatory action	Mu et al. 2009
Baicalein	10–50 μM	Thapsigargin and brefeldin A-induced HT22 mouse hippocampal neuronal cells	Attenuating sub-G1 fractions from 55.27–63.84% to 26.20–28.96%	Reducing CHOP induction and ROS accumulation and mitochondrial damage.	Choi et al. 2010
Baicalein	1, 2 and 4 mg/kg for 1 time (acute) and 21 d (chronic), i.p.	Acute and chronic depression rats	Reducing the immobility time in the forced swimming test and tail suspending test	Hippocampal ERK-mediated neurotrophic action	Xiong et al. 2011
Baicalein	1–30 μM	α-*syn*-Oligomer-induced SH-SY5Y cell toxicity, Aβ fibril-induced PC12 cells toxicity	Protection rate, 62–80%	Inhibiting formation of α-synuclein pligomers within living cells and prevents Aβ peptide fibrillation and oligomerization	Lu et al. 2011
Baicalein	140, 280 mg/kg for 7 d, i.g.	MPTP-induced Parkinson's disease in mice	Shortening the total time for climbing down the pole, prolonging the latent periods of rotarod, and increasing the vertical movements	Regulation of genes such as LIMK1, SNCA and GLRA1	Gao et al. 2015
Baicalein	1 mg/kg pretreated for 2 (1st and 4th) or 4 times, i.p.	Methamphetamine-induced amnesia in ICR mice	Attenuating memory deficits and oxidative hippocampal damage		Wong et al. 2014
Baicalein	200, 400 mg/kg for 28 d, p.o.	Rotenone-induced Parkinson's disease in SD rats	Attenuating behavioral impairments and the depletion of dopaminergic neurons; restoring mitochondrial function and improved mitobiogenesis	Through the cAMP-responsive element binding protein (CREB) and glycogen synthase kinase-3β (GSK-3β) pathways	Zhang X et al. 2017a
Baicalein	200, 400 mg/kg for 28 d, p.o.	Rotenone-induced Parkinsonian SD rats	Improving motor impairments, attenuateing brain damage, suppressing the production of proinflammatory cytokines, modulating the astrocytes and microglia activation	Through anti-neuroinflammation	Zhang X et al. 2017b
Baicalein	30 mg/kg for 4 d, i.p.	Acrolein-induced Parkinsonian SD rats	Attenuating oxidative stress and protein conjugation and inhibiting inflammation in the nigrostriatal dopaminergic	Inhibiting oxidative stress, protein conjugation, and inflammation	Zhao WZ et al. 2018
Baicalin	1–100 μM	Prolyl oligopeptidase	IC_50_, 12 μM		Tarragó et al. 2008
Baicalin	0–10 μM	Aβ 1–42-induced SH-SY5Y cell injury	Protecting cells viability from 57% to 78% at high dose	Inhibiting Aβ 1–42 aggregation and reducing H_2_O_2_-mediated oxidative stress and damage	Yin et al. 2011
Baicalin	100, 200 mg/kg for 7 d, i.p.	Transient global cerebral ischemic-reperfusion injury in Mongolian gerbils	Attenuating ischemia-induced neuronal cell damage	Related with anti-oxidative and anti-apoptotic properties	Cao et al. 2011
Baicalin	100 mg/kg of twice /day for 7 d, i.g.	Global cerebral ischemia/reperfusion rats	Improving the learning and memory	Inhibition of COX-2 expression	Cheng OM et al. 2012
Baicalin	200 mg/kg/day for 7 d, i.g.	Transient global cerebral ischemia Mongolian gerbils	Facilitating neurological function, suppressing the ischemia-induced neuronal damage	Activating GABAergic signaling, HSP70 and MAPKs cascades in global ischemia	Dai et al. 2013

Baicalein (2 μM) could significantly promote mouse hippocampal HT22 cell survival by 50% after injury induced by iodoacetic acid (Lapchak et al. 2007). Baicalin (200 mg/kg for 7 d, i.p.) attenuated neurological impairment in gerbils after global ischemia, reducing neurological deficit scores from 2.88 to 1.63 (Dai et al. 2013). Baicalin could also protect against neuronal loss and apoptosis in gerbil hippocampus by activating GABAergic signaling, HSP70 (70 kilodalton heat shock proteins), and MAPKs cascades (Dai et al. 2013). The neuro-protective activities of *S. baicalensis*, baicalein, baicalin, and wogonin indicate they may be promising neuroprotective agents for the prevention of Alzheimer’s disease, Parkinson’s disease, ischemic strokes, and other neurologic diseases (Cho and Lee 2004; Li et al. 2005; Cheng et al. 2008; Tarragó et al. 2008; Mu et al. 2009; Choi et al. 2010).

### Hepatoprotective activities

Extracts of *S. baicalensis* could inhibit liver injury and fibrosis in BDL (bile duct ligation), CCl_4_, and LPS-induced rat or mouse hepatotoxity, by inhibiting cytokine, COX-2, iNOS (nitric oxide synthases), and NF-κB (Nan et al. 2002; Thanh et al. 2015). Baicalin (5 mg/kg for 5 d, i.p.) could protect against *t*-BHP-induced rat liver injury, reducing the ALT (alanine transaminase) and AST (aspartate transaminase) levels from 226 to 110 U/l and from 607 to 197 U/l, respectively (Hwang et al. 2005). Baicalin also exerted hepatoprotective effects in alcohol-induced liver injury through inhibiting oxidative stress, inflammatory response, and regulation of the Shh pathway (Wang HF et al. 2016).

### Effects on cardiovascular and cerebrovascular diseases

Baicalin (6 μM) could protect against the hyperglycemia-induced cardiovascular malformation during chick embryo development, decreasing the high incidence of cardiac bifida from 32% to 16%, by reducing ROS production and regulating SOD (superoxide dismutase), GSH-Px (glutathione peroxidase), and GABA_A_ (γ-aminobutyric acid) levels (Wang et al. 2018). Baicalin also exerted angiogenesis and cardioprotective effects against chronic hypoxia-induced pulmonary hypertension and acute myocardial infarction *in vivo*, through mediation of MAPK cascades, the ERRα (estrogen-related receptor α) pathway, and the PI3K/AKT signaling (Zhang et al. 2011; Liu et al. 2013; Huang et al. 2017).

Baicalein could promote new blood vessel formation, attenuate cardiac remodeling and endothelium dysfunction against angiotensin II or myocardial ischemia reperfusion injury, via inhibition of AKT/mTOR, ERK1/2, NF-κB, and calcineurin sgnaling pathways in mice or chicks (Cho et al. 2008; Li et al. 2015; Wang AW et al. 2015).

### Effects on bones

The extract of *S. baicalensis* (50 mg/kg for 42 d, p.o.) could significantly increase bone mineral density by 12–18%, and improve bone trabecula microstructure of weightlessness induced osteoporosis rats via the osteogenic differentiation enhancement effect (Zhang GW et al. 2017). A wogonin-rich fraction (50 μg/mL) exerted chondroprotective effects by inhibiting ROS production and suppressing catabolic markers (Khan et al. 2017). Baicalein and baicalin (10 μM) could significantly enhance the osteogenic differentiation of human periodontal ligament cells (hPDLCs) and rat bone marrow derived mesenchymal stem cells (rBMSC), reapectively, by increasing ALP (alkaline phosphatase) activities up to 1.5-2-fold and increasing the formation of mineralized nodules up to 2-fold (Chen et al. 2017; Zhang GW et al. 2017). Arjmandi et al. (2014) reported that UP446 (a natural proprietary of *S. baicalensis* and *Areca catechu* L.) could reduce physical symptoms associated with knee osteoarthritis in patients after 500 mg/d treatment for 1 week.

### Other activities

The extract of *S. baicalensis* and baicalein (1 mg/kg and 1 μg/kg for 2 d, respectively, p.o.) could reduce gastrointestinal dysfunction in ritonavir-treated rats (Mehendale et al. 2007). An ethanol extract exerted synergistic anti-diabetic effect with metformin in STZ-induced diabetic rats. Baicalin possessed anti-hyperglycemic activities by suppressing hepatic gluconeogenesis (Waisundara et al. 2008; Wang T et al. 2017). Baicalein could reduce endometriosis by suppressing the viability of human endometrial stromal cells *in vitro* (Jin et al. 2017). Furthremore, baicalin exhibited embryo-protection (Qi et al. 2016), weight losing (Yun and Jung 2014), sleep–wake regulation (Chang et al. 2011), anti-allergic (Kim et al. 2010), and anti-pyretic effects (Tsai et al. 2006). The polysaccharides from *S. baicalensis* showed antioxidative and immunostimulating activities (Olennikov et al. 2008a, 2011).

## Biosynthesis of *Scutellaria* flavonoids

**Figure 5.**
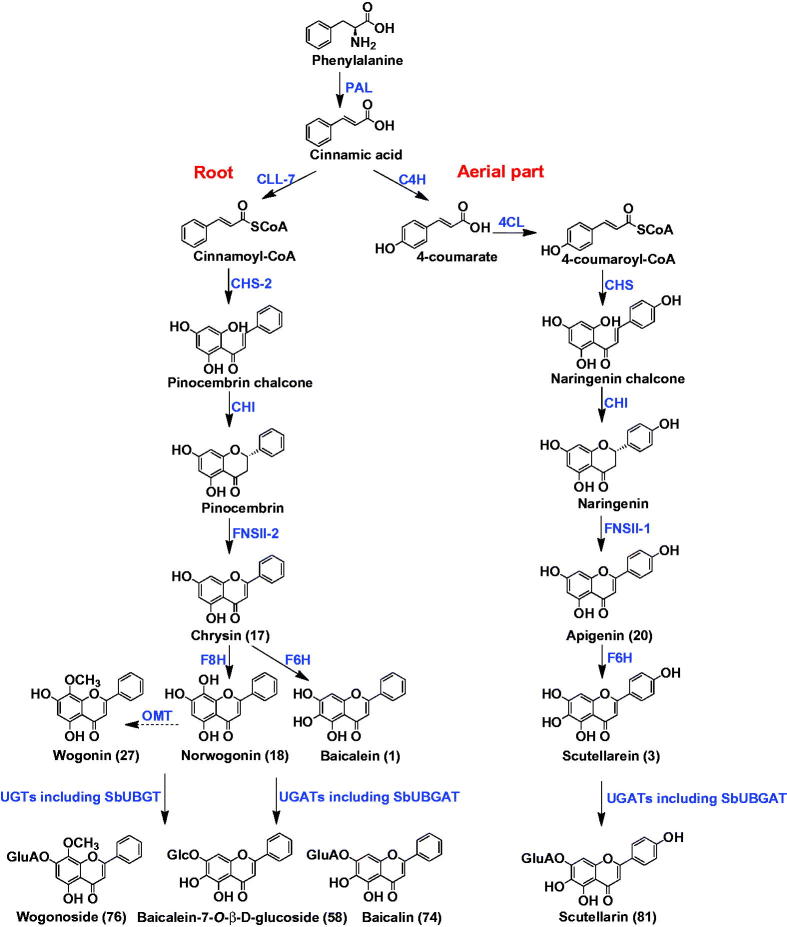
Different biosynthesis pathways in roots and aerial parts of *Scutellaria baicalensis*.

The flavonoids in *S. baicalensis* Georgi possess various pharmacological activities. Their biosynthesis in the living plant has gained increasing attention in recent years. Zhao et al. systematically investigated the biosynthetic pathways of free flavones. The *Scutellaria* flavones are originally derived from phenylalanine, which is catalyzed by phenylalanine ammonia lyase (PAL) to form cinnamic acid. Interestingly, the subsequent biosynthetic steps were different for flavones in the aerial parts and in the roots (Figure 4). For the 4′-hydroxyl flavones, which are mainly distributed in the aerial parts, cinnamic acid is sequentially catalyzed by cinnamoyl 4 hydroxylase (C4H), *p*-coumaroyl CoA ligase (4CL), chalcone synthase (CHS), chalcone isomerase (CHI), and flavone synthase (FNSII-1) to form apigenin (Zhao et al. 2016a, 2016b). Then apigenin is hydroxylated by flavone 6-hydroxylase (F6H) to generate scutellarein, as shown in Figure 5 (Zhao Q et al. 2018). The flavones in the roots, however, usually lack a 4′-OH group on the B-ring. For their biosynthesis, cinnamic acid is catalyzed by cimmamoyl-CoA ligase (CLL-7), chalcone synthase (CHS-2), chalcone isomerase (CHI) to form pinocembrin. Pinocembrin is then converted by a specialized isoform of flavone synthase (FNSII-2) to form chrysin, which could be further hydroxylated by flavone 6-hydroxylase (F6H) and flavone 8-hydroxylase (F8H) to produce baicalein and wogonin, respectively (Zhao et al. 2016a, 2016b, 2018). *O*-methyltransferases (OMTs) may participate in the biosynthesis of wogonin, though no OMT has been reported yet. Among the biosynthetic enzymes, SbCLL-7, SbCHS-2, FNSII-2 and F8H are expressed preferentially in the roots. Functions of these genes have been validated by RNAi in hairy roots of *S. baicalensis* and overexpression in transgenic *Arabidopsis*.

**Figure 6.**
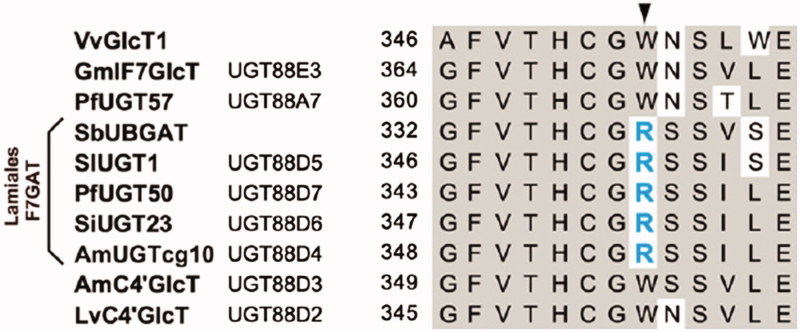
Key amino acid residues for the catalytic selectivities of *O*-glucuronyltransferases and *O*-glycosyltransferases (Adapted from Noguchi et al. 2009).

Glycosyltransferases are responsible for the formation of glycosidic bonds of flavonoid *O*-glucuronides and *O*-glucosides of *S. baicalensis*. SbUBGAT showed *O*-glucuronyltransferase activities for various flavones, and may take part in the biosynthesis of glucuronides like baicalin and wogonoside (Nagashima et al. 2000; Yang et al. 2016). SbUBGAT also showed *O*-glycosyltransferase activities. Together with SbUBGT discovered from the hairy root cultures of *S. baicalensis*, they may contribute to the production of flavonoid-*O*-glycosides (Hirotani et al. 2000). Furthermore, the Arg residue (R) in the PSPG (Plant Secondary Product Glycosyltransferase) box plays a critical role in the recognition of UDP-glucuronic acid sugar donor, while the corresponding Trp residue (W) has better selectivity for UDP-glucose donor (Figure 6). This was validated by homology-modeling and site-directed mutagenesis analysis (Noguchi et al. 2009). *Scutellaria baicalensis* also contains abundant flavonoid-di-*C*-glycosides, and the responsible *C*-glycosyltransferases have not been reported yet.

## Conclusions and future prospects

*Scutellaria baicalensis* contains at least 126 small molecules and 6 polysaccharides. It possesses anti-tumor, anti-viral, anti-microbial, anti-inflammatory, antioxidative, and neuroprotective activities. Chemical compounds responsible for many of these activities are still unknown, though the bioactivities of a few major compounds (baicalin, baicalein, wogonoside, and wogonin) have been extensively studied. Recently, our group reported the comprehensive correlations of chemicals and bioactivities of another popular herbal medicine Gan-Cao (licorice, *Glycyrrhiza uralensis* Fisch), and discovered a number of promising bioactive natural products (Ji et al. 2016). Similar research strategy could be applied to Huang-Qin to discover potential new drugs. In fact, the clinical trial of wogonin as an anti-cancer drug candidate has recently been approved by the State Drug Administration of China. On the other hand, the identified major bioactive compounds could be used as chemical markers to improve quality control of Huang-Qin crude drugs and related patent drugs. Furthermore, biosynthetic studies could help large-scale production of the bioactive compounds by metabolic engineering. Although enzymes involved in the biosynthesis of free flavones have been reported for *S. baicalensis*, many post-modification enzymes have yet to be characterized, including those responsible for the hydroxylation, methylation, and glycosylation reactions.
